# Factors determining the dorsal coloration pattern of aposematic salamanders

**DOI:** 10.1038/s41598-022-19466-0

**Published:** 2022-10-12

**Authors:** Benedetta Barzaghi, Andrea Melotto, Paola Cogliati, Raoul Manenti, Gentile Francesco Ficetola

**Affiliations:** 1 Department of Environmental Science and Policy, University of Milano, Milan, Italy; 2 Centre of Excellence for Invasion Biology, Department of Botany and Zoology, Stellenbosch University, Stellenbosch, 7600 South Africa; 3 Laboratoire D’Ecologie Alpine (LECA), CNRS, Univ. Grenoble Alpes, Grenoble, France

**Keywords:** Ecology, Evolution

## Abstract

Aposematic bright colors have a key role for animal defense and can be expressed through metabolic production or by acquiring pigments from diet. Aposematic coloration can be related to both local adaptations and availability of trophic resources. The European fire salamander (*Salamandra salamandra*) shows significant color variability and occurs across a broad range of habitats. Here we combined field observations with common rearing experiments to disentangle the role of environmental conditions and local adaptations in determining aposematic coloration of salamander populations. We assessed color variation and measured habitat features and food availability in adults from 25 populations. Furthermore, we reared newborn larvae from 10 populations under different food availability and analyzed color of metamorphs. To assess color pattern, we measured the percentage of yellow covering the body, and the Hue, Saturation and Value of yellow coloration. Adult showed strong variation of color pattern; variation was strongly related to the individual's size, to habitat productivity and to food availability. Under common garden conditions, differences between populations were not anymore evident, and coloration was only affected by resource availability during larval development. Our results suggest that environmental conditions and food availability are more important than local adaptations in determining differences in aposematic color pattern.

## Introduction

Variation of animal coloration has always caught the attention of naturalists^1–3^. The huge intra- and inter-specific variation of animal coloration is determined by the joint effect of multiple forces, including physiological constraints, sexual selection and natural selection^4–6^. Aposematic organisms have defensive traits that make them unpalatable, and display warning signals to advertise these defenses to potential predators^7–9^, thus they are among the animals with the most striking coloration. The aposematic strategy relies on the ability of the predator to recognize the alarm signal and associate it to the unpalatability of prey^9^. Since predators are faster to learn and to avoid the aposematic prey when this signal is as unique as possible, theory predicts that aposematic species should be under strong stabilizing selection, resulting in monomorphism^10^. The presence of polymorphic warning signals in aposematic taxa, therefore, represents an evolutionary paradox due to the presumably higher cost of predator education^11,12^.

Nevertheless, intraspecific variation and polymorphism of aposematic coloration are widespread, and evolutionary, ecological and physiological processes have been proposed to affect this variation^13–15^. First, the expression of aposematic colorations may often represent a non-negligible cost. The development of the yellow-orange-red coloring typical of aposematic patterns in vertebrates is generally linked to different typologies of pigments such as carotenoids, riboflavin and pteridines^16^. Carotenoids generally require to be gathered through foraging and have limited availability in certain environments^17^, while pteridines have a significant cost of synthesis^18,19^. The costs of the production and maintenance of effective warning signals can reduce longevity in butterflies^20^, impact the growth rate in ladybird beetles^21^, and reduce immune defenses in multiple animal species^22^. Therefore, spatial variation in aposematic coloration can be determined by the availability of resources required to produce pigments. Second, aposematic animals can use their bright coloration also for sexual or social signaling, thus sexual and social selection can concur in determining variation of aposematic coloration^12,23,24^. Third, aposematism can interact with additional functions of coloration, such as crypsis or thermoregulation^25,26^. For instance, in the aposematic tiger moths, individuals living at higher latitudes are darker; this improves their thermoregulation performance but reduces defensive effects against predators^27^. Fourth, spatial variation in the abundance or diversity of predators can determine different needs of aposematic signaling. For instance, individuals in areas with higher predator abundance could be under stronger frequency-dependent selection, or could show brighter aposematic coloration^13,28^. Finally, coloration can covary with additional features of individuals (e.g. age, body size, expression of additional defenses…), further complicating the pattern of color expression and variation in aposematic species.

The multiple potential drivers of intraspecific aposematic variation (e.g. resource availability vs. selective pressures) make it challenging identifying the underlying processes. Some populations can display brighter colors because of a better availability of resources (phenotypic plasticity), or because of spatial variation of selective forces (adaptive variation). Coloration plasticity is the environmentally-induced colour variation on a certain genotype and it is a widespread phenomenon. For instance, in *Ambystoma* salamanders, early-stage larvae can plastically change their color because of variation in temperature conditions, while older larvae do not, likely as an adaptation to the environment in which they metamorphose^29^. Furthermore, the ability to modify colour in response environmental variation may itself be considered an adaptation. Rearing individuals under common environmental conditions is an excellent strategy to tease apart the role of plastic vs adaptive variation, and the results of common garden experiments can be integrated with data on wild populations to better identify the drivers of spatial variation. While the role of phenotypic plasticity and local adaptions is widely investigated, so far very few studies considering aposematic species combined both field data and cross environment experiments to identify the drivers of intraspecific variation of aposematic coloration^30^.

Aposematic amphibians are excellent model species to understand the relative role played by adaptation and plasticity on variation of aposematic patterns. Many species are toxic and aposematic and show impressive cases of both local adaptations and phenotypic plasticity in different environmental conditions^17,31^. In this study we used a widespread, aposematic, European amphibian, the fire salamander (*Salamandra salamandra*, considering just one lineage) to understand the relative role played by trophic resources availability and potential selective forces on the variation of aposematic coloration. Spatial variation of salamander coloration is well known^32,33^, but very limited information exists on whether these differences are determined by plastic variation or local adaptations.

First, we compared the aposematic coloration of adults from multiple populations inhabiting very different environments, to test whether they show significant intraspecific variation and to identify the spatial drivers of such variation. Specifically, we evaluated whether color differences between populations are related to spatial variation of resources, habitat productivity and predation risk. Second, we used a common garden experiment to compare the variation in post-metamorphic coloration of larvae reared under different conditions of trophic resources availability. If color variation across populations is mainly determined by local adaptation to the extant selective pressures, we predict to detect differences similar to the ones observed in wild-caught adults. This independently on the rearing conditions experienced during the common garden experiment. Conversely, if color variation is mostly a plastic response to the experienced environmental conditions, we expect the color of larvae reared in common garden experiments to depend on resources availability, irrespective on population of origin.

## Methods

### Study species, area, and sampling strategy

The fire salamander is a eurythermal and eurizonal species, and its distribution ranges from the sea level to 2000 m a.s.l. in the Alps, with maximum frequency between 200 and 700 m a.s.l.^34^. This species shows a high intraspecific plasticity for ecology, developmental features, morphological traits and reproductive strategy^35,36^. The fire salamander is ovoviviparous and typically breeds in small streams and headwaters, but some populations also use different water bodies typologies, such as temporary and permanent mountain pools, artificial ponds and groundwaters^37,38^. The study area is located between the districts of Como, Lecco and Monza-Brianza (Lombardy NW Italy: approx. 45.9 N, 10.0 E). In this area, the fire salamander is widely distributed in broad-leaved forests at altitudes between 300 and 700 m a.s.l., where it is common in the surroundings of its breeding habitats, such as small streams, springs and ponds^37^. However, within this area high altitude populations are not rare, with a maximum altitude record of to 1491 m a.s.l.^35^. Variation in altitudinal range is expected to determine different selective pressures on salamanders because both trophic resources and predators are likely less abundant at higher altitudes^39,40^. For the present study, we thus identified sampling sites covering the whole altitudinal range of the species (i.e., 250–1491 m a.s.l.) where we performed (1) night surveys of to assess variation in aposematic of coloration of adult salamander; and (2) larvae collection at breeding sites to rear them under different nutritional regimes. All the study populations belong to the same subspecies (*Salamandra salamandra salamandra*) and to the same ecotype.

### Adults samplings and characterization of trophic availability, predation risk and ecosystem productivity

From October 2017 to October 2018 we performed night sampling at the different 25 populations. All populations were > 5 km apart; available studies showed very limited gene flow in fire salamander populations at distances > 1 km^41^. For each site we surveyed a 100-m transect along which we collected all the adult salamanders that we encountered. Two successive surveys were performed for each transect during the same night. If in a site we did not detect salamanders we repeated the survey at least two times during successive nights with appropriate weather conditions (rain or high humidity); overall, we found adult salamanders in 24 sites (see Supplementary Table S1). All the collected salamanders were photographed (see below) and immediately released.

In order to assess the prey availability for adult salamanders, in each terrestrial site after the second survey we counted the number of earthworm droppings. To count earthworm droppings, we placed along each transect, about every 25 m, a square (40 cm × 40 cm; in total 4 squares surveyed in each transect) in which we counted the number of droppings. Given that earthworms activity is strongly affected by weather conditions, we retained the highest number of droppings detected in each transect for analyses. Preliminary tests revealed that there is a strong correlation between the number of droppings at the surface and the density of earthworms in the soil (Manenti and Barzaghi, unpublished). Results remained consistent if the average number of droppings is considered instead of the maximum.

We used citizen-science data to assess the diversity of predators, as predators could affect aposematic coloration. Strigiformes (owls; *Athene noctua*, *Aegolius funereus*, *Tyto alba*, *Strix aluco* and *Asio otus*) and grass snakes (*Natrix natrix*) are the major predators of adult salamanders in the study area^42,43^. Predator richness in study sites was estimated using the online database Ornitho (www.ornitho.it). Ornitho is a citizen-science gateway collating ~ 19,000,000 occurrence data of vertebrates. Using the default resolution available in Ornitho, we counted the total number of predator species within the 10 × 10 km UTM square where each population is located. By integrating data on species ecology (e.g., occurrence of habitats) and distribution it is possible to obtain accurate inference on trophic links occurring in reality^44^. All the sites we surveyed have suitable habitats for the owl species present in the respective squares, while the grass snake is rarer and generally occurs below 1000 m a.s.l.^45^. Finally, for each site we used the Normalized Difference Vegetation Index (NDVI) as a measure of primary productivity of ecosystems. NDVI is a proxy of photosynthetic activity and green biomass and can be related to the amount of resources available for animals^46^. NDVI was obtained from 1-km resolution SPOT-VEGETATION data from the European Space Agency (http://maps.elie.ucl.ac.be/CCI/viewer/download.php).

### Larvae sampling and rearing

From March 2017 to May 2017, we collected 10 newborn fire salamander larvae from 15 sites (total: 150 larvae; see Supplementary Table S1). This variation in larvae collection period (53 days in total) is due to different times of deposition between mountain and foothill populations^47^. We collected newborn larvae to minimize the possible influence of environmental conditions in breeding sites. Immediately after the collection, each larva was placed in a transparent plastic tank (size 79 × 57 × 18 cm/55 l) and photographed on graph paper to allow body size measurement and subsequent individual identification. At the time of capture, salamander larvae had an average (± SE) total body length of 33.66 mm (± 0.26 mm); preliminary analyses using linear mixed models did not detect any significant correlation between the size of newborn salamanders and the altitude of populations (Supplementary materials S1). We randomly divided the 10 larvae from each site in two treatment groups (5 individuals each): “poor nutritional condition” and “rich nutritional condition”. The larvae with poor food conditions were given a single chironomid larva every second day. Instead, salamander larvae assigned to rich food condition were fed daily with ad libitum chironomid larvae. Chironomid larvae are generally rich in different types of carotenoids^48^ that amphibians acquire through diet and that may affect color patterns of aposematic species^17^.

Larvae were kept separated in plastic cups (diameter 8 cm) that were placed in four plastic tanks (80 cm × 70 cm), filled with 20 L of tap water. Individual plastic cups were perforated and each of the four plastic tanks hosted both larvae with poor and rich food conditions. The position of cups containing larvae within the tanks was randomized and randomly changed every week during the treatment. During rearing water temperature (16 °C) and oxygenation were maintained constant and water was regularly cleaned of metabolic residues and monthly replaced. The substrate was the same for all the larvae (clear plastic bottom of the tank).

After metamorphosis salamanders were kept under rearing conditions for 45 days before analysing their aposematic coloration. Young salamanders were individually housed in terrariums (length 38 cm, width 28 cm, height 18 cm), marked with the identity code and metamorphosis date. In each terrarium salamanders were provided with a moistened broadleaved wood substrate, to mimic natural conditions. We fed individuals twice a week with 2 cm long earthworms, by slowly moving them for two minutes in front of the salamander. Once the study was completed, the young individuals of fire salamander were released in their sites of origin. Overall, the rearing experiment lasted around 16 months: from March 27th 2017 to July 14th 2018.

### Photo and color analysis

**Figure 1:**
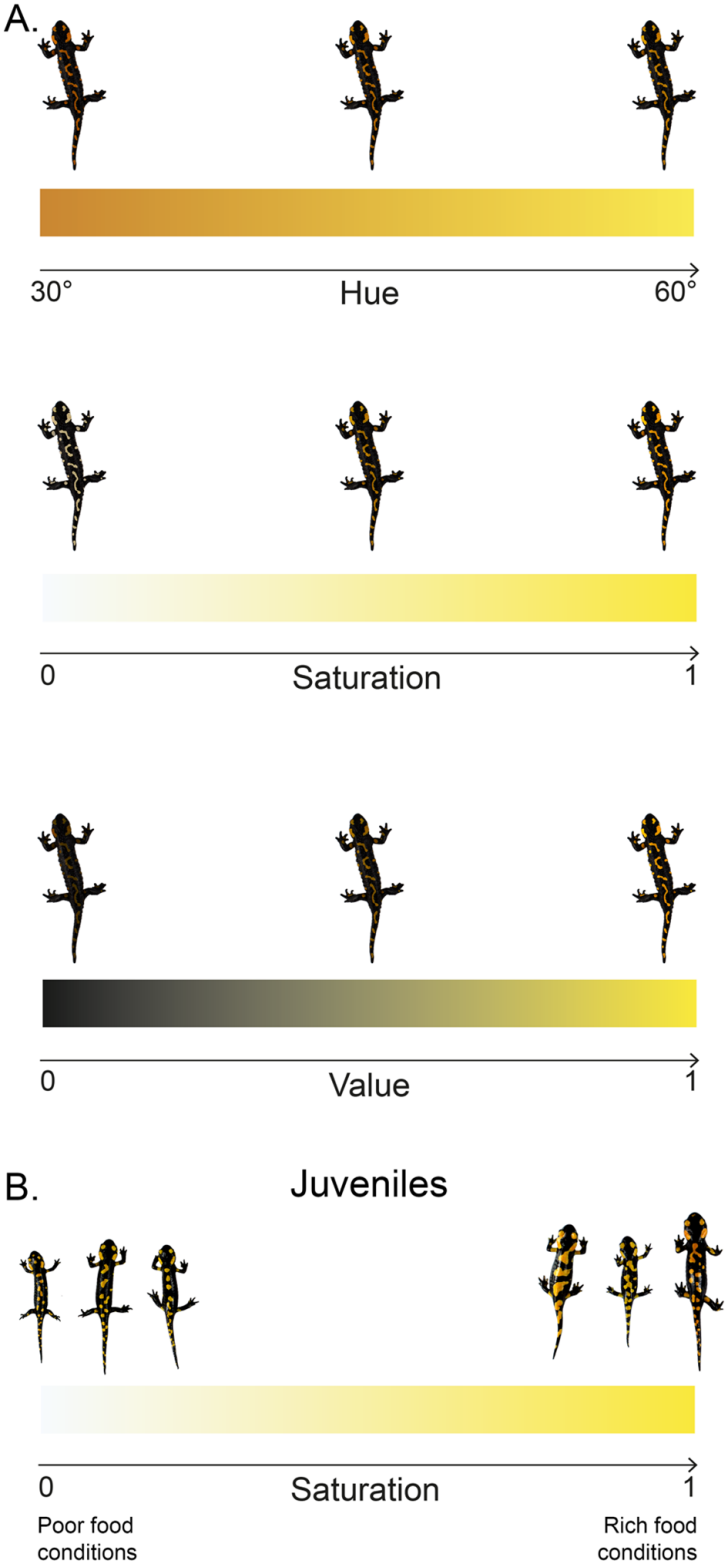
Demonstration of how the variation of Hue Saturation Value (HSV) can affect the color of salamanders. (**A**) Example of variation in a hypothetic adult salamander. (**B**) Real variation observed in juveniles reared in poor and rich food conditions.

The dorsal photos, of both adults and metamorphs were taken in a softbox illuminated with four led spotlights (color temperature 4000 K). Photos were taken with a Canon 700D SLR using a Canon 18-55 lens. In order to standardize pictures, a white balance was taken using a Pantone © colorimetric scale as reference (model: color checker "X-Rite Standard Passport"). Each photograph was taken in manual mode (settings: focal length 35 mm, f/10.0, shutter speed 1/200 and ISO 100). Each photograph was taken in RAW mode to limit loss of detail. For each photograph the salamander was placed in the center of the softbox on a white background provided with a colorimetric scale and a millimeter scale to allow size and color measurements. The camera was attached to a Manfrotto photographic tripod which was positioned directly above the softbox to hold the lens at 45 cm from the base on which the salamanders were positioned. The camera was kept horizontal with the help of the app "level" present in the iPhone 6S. For each image we used the raw file and we made white balance at three levels; 3 pixels were selected, the blackest, the whitest and the medium gray pixel, on the basis of the Pantone using Adobe Photoshop CC2017 version. After white balancing, we used the "quick selection" tool set to 20 to outline the salamanders and create a new layer containing only the individual. Subsequently, using the "color range" tool, each yellow spot on the salamander was selected. We then used the “dropper” tool to select all the yellow pixels, starting from a yellow pixel in the central part of the salamander back and with a tolerance of 150. The selected yellow pixels were then used to calculate the percentage of yellow of each individual. Subsequently jpg files containing only the selected yellow pixels were imported in the R environment and using the *Raster*, *Jpeg* and *Colorspace* packages to extract the HSV (Hue, Saturation and Value) scores of all the yellow pixels of all the individual pictures. The HSV model is an approach of color measurements that is alternative to the RGB model (Fig. 1). The Hue (or tonality) which ranges from the primary red (0°), to primary green (120°), primary blue (240°), and then back to the red (360°). The Saturation (purity) is the intensity and purity of the colour; while the Value (brightness) is an indication of its brilliance; both saturation and brightness are percentages.

### Statistical analyses

We used a combination of multivariate and univariate models to identify the factors influencing the aposematic yellow coloration of both metamorphosed reared larvae and adults sampled in the same sites of origin. For both new-metamorphosed and wild-collected adults, independent variables included factors representing trophic availability, altitude and predator richness in the site of origin and features of the individuals (i.e., body size and sex for the adults).

For the analysis of wild-collected adult salamanders, we conducted two multivariate analyses of variance (MANOVA), considering as dependent variable the extracted components/attributes of the yellow coloration (percentage of yellow and average values for Hue, Saturation and Value). First, we run a MANOVA including only population identity as independent variable in order to test whether populations actually show significant color differences. Then, we run a second MANOVA to identify the environmental factors related to such differences. In this MANOVA, the four-color variables were included as dependent, while prey availability, NDVI, altitude, and richness of predators were fixed factors and population identity was included as random factor. Both multivariate models included individual sex and total length as covariates as these features can affect coloration. Linear mixed models (LMMs) were then used to assess the relationship between each attribute of the yellow coloration (percentage of yellow, hue, saturation, value) and the environmental factors included in the MANOVA; population identity was included as an additional random factor in LMMs. We assessed the significance of the factors composing LMMs for each yellow component using a F-test with Satterthwaite approximated degrees of freedom.

We used a similar approach for larvae. First, we ran a MANOVA with the components of the yellow pattern as dependent, population identity and rearing conditions to assess coloration variation among population or different nutritional regimes as independent variables. We then ran a second MANOVA considering as independent variables rearing conditions and environmental features of the population of origin (altitude, NDVI, prey availability, and the richness of predators in the site of origin). In this multivariate analysis, the length of individuals at metamorphosis and population of origin were respectively included as additional covariate and random factor. The MANOVA was then followed by a separate LMMs for each of the four yellow attributes using the same fixed and random factors included in MANOVA. All analyses were carried out in the R environment, using the *lme4*^49^ and *lmerTest*^50^ packages, while *visreg*^51^ was used to produce conditional regression plots.

### Ethics declarations

The animal study protocol was approved by the Institutional Review Board (or Ethics Committee) of LOMBARDY REGION AUTHORITY (T1.2016.0052349. of December 10 2016). All experiments were performed in accordance with the approved protocol and with the ARRIVE guidelines.

## Results

### Yellow pattern in wild-caught adults

We analyzed the yellow pattern of 283 males and 180 females. Their average (± SE) total length was 16.7 ± 3.09 cm with males ranging from 10.45 to 25.08 cm and females ranging from 11.06 to 23.42 cm; the average percentage of dorsal surface covered by yellow coloration was 14.41% ± 0.6 and ranged in males between 0.6 and 65% while in females ranged from 0.2 to 79%. Averages (± SE) of color attributes were 0.13 ± 0.02 for Hue, 0.76 ± 0.1 for Saturation and 0.63 ± 0.12 for Value. In males Hue ranged between 0.04 and 0.20, while in females from 0.09 to 0.23. Saturation in males ranged between 0.40 and 0.94 and in females between 0.34 and 0.91. Value ranged from 0.33 and 0.99 in males and 0.36 and 0.99 in females. The MANOVA assessing the differences across populations revealed a substantial variability of the yellow pattern between sites (MANOVA, Pillai’s trace 13.6, *P* < 0.001). The subsequent MANOVA including environmental features showed a strong relationship between color pattern, prey availability and productivity (NDVI) (MANOVA: all *P* ≤ 0.03 in the between-sites analysis, Table 1).

**Table 1:** Multivariate analysis of variance (MANOVA) assessing the factors determining the variation of salamander’s dorsal pattern (hue, saturation, value and percentage of yellow) in adult, wild-caught individuals.

Independent variable	Pillai’s trace	d.f.	F	*P*
**(a) Between populations**				
Prey availability	0.686	4,13	7.10	**0.002**
Sex	0.782	4, 13	11.67	** < 0.001**
Total length	0.32	4, 13	1.53	0.250
NDVI	0.579	4, 13	4.47	**0.017**
Altitude	0.336	4, 13	1.64	0.221
Predator richness	0.19	4, 13	0.76	0.565
**(b) Within population**				
Prey availability	0.02	4, 433	2.31	**0.057**
Sex	0.011	4, 433	1.26	0.28
Total length	0.055	4, 433	6.35	** < 0.001**
Altitude	0.007	4, 433	0.83	0.50

The MANOVA included population identity as random factor, therefore distinguished between factors determining differences between and within populations. Note that individuals from the same population can be collected in areas with different altitude and prey availability (prey availability was measured in three sites per each population). Significant effects are in bold. TL, total length; NDVI, normalized difference vegetation index.

**Figure 2:**
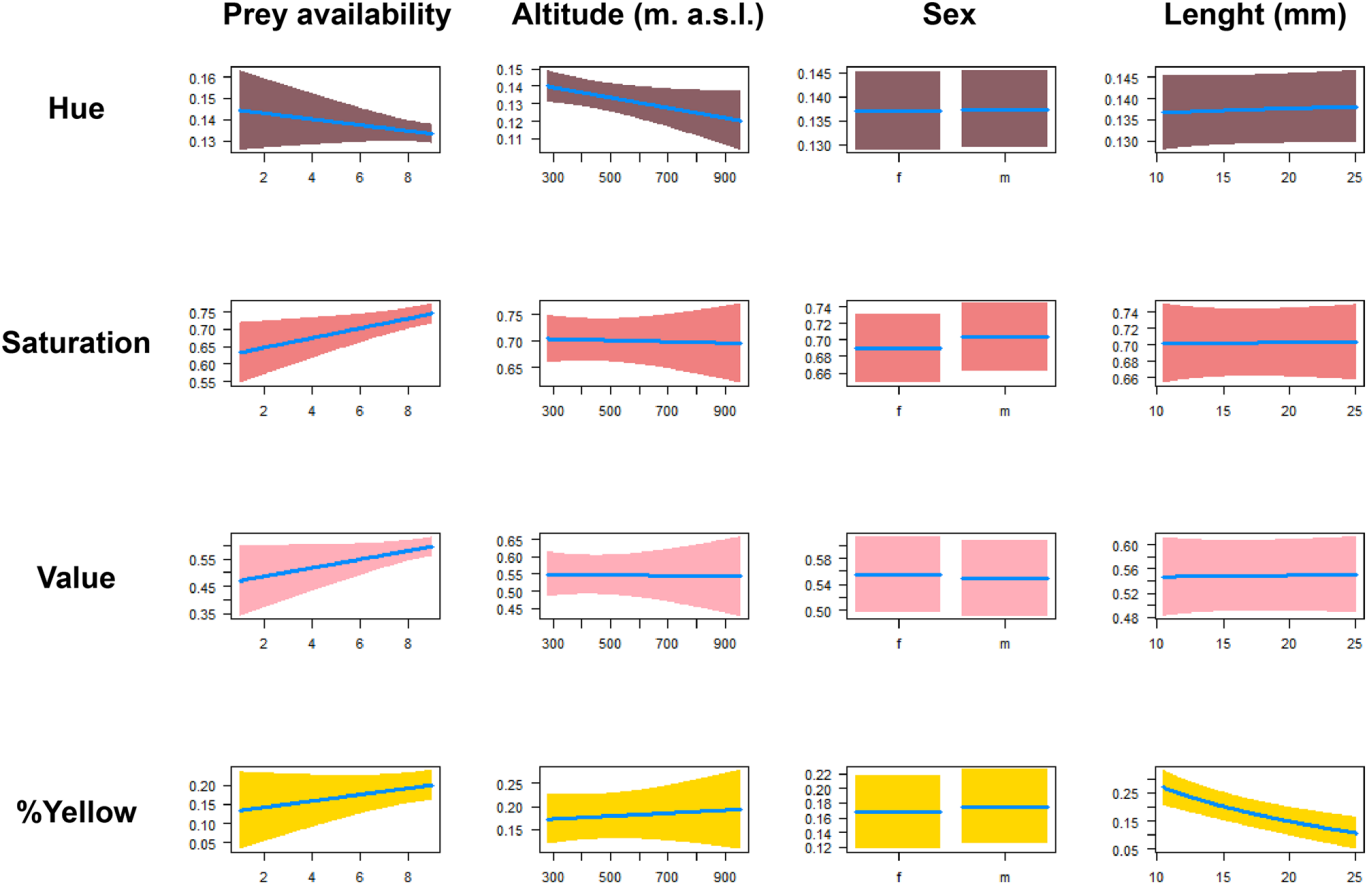
Conditional regression plots showing the results of univariate models (LMMs) assessing effect of fixed factors on components of the yellow coloration. Coloration components are tonality (Hue), saturation (Sat), brightness (Value) and the percentage of yellow coverage. The effect of the prey availability, the altitudinal range and the length of the salamander and the sex are shown (for a complete description of univariate model results see Table 1). The blue line represents the estimated median, while colured areas are 95% confidence bands.

Univariate analyses showed that the four parameters of coloration are differently affected by fixed factors we tested. The percentage of yellow coloration was strongly related to the length of individuals; shorter individuals have a higher proportion of yellow (Table 2, Fig. 2). Hue component was significantly lower in individuals from areas with higher prey availability, and in populations with increased productivity values. Saturation and Value components only showed a weak relationship with the availability of prey, with a tendency to be higher in sites with higher earthworm density (Table 2, Fig. 2).

**Table 2:** Univariate linear mixed models (LMMs) assessing the factors determining the variation of components of salamander’s dorsal pattern (hue, saturation, value and percentage of yellow) in adult, wild caught individuals.

Dependent variables	Independent variables	Effect	NumDF	DenDF	F	*P*
Percentage of yellow	Prey availability	+	1	18.08	1.05	0.319
	Sex (male)	+	1	454.78	0.45	0.50
	TL	−	1	455.22	22.29	** < 0.001**
	NDVI	−	1	15.71	0.51	0.485
	Altitude	+	1	16.45	0.07	0.782
	Predator richness	+	1	15.06	0.11	0.741
Hue	Prey availability	−	1	33.69	0.9	0.347
	Sex (male)	+	1	442.20	0.1	0.745
	TL	+	1	443.83	0.18	0.669
	NDVI	−	1	17.87	7.30	**0.014**
	Altitude	−	1	19.38	3.01	0.098
	Predator richness	+	1	17.78	5.67	**0.028**
Saturation	Prey availability	+	1	23.90	5.78	**0.024**
	Sex (male)	+	1	451	2.4	0.121
	TL	+	1	454.44	0.002	0.962
	NDVI	+	1	19.06	0.002	0.962
	Altitude	−	1	20.04	0.02	0.877
	Predators	−	1	18.59	3.54	0.075
Value	Prey availability	+	1	27.67	3.19	0.084
	Sex (male)	−	1	445.32	0.32	0.566
	TL	+	1	447.96	0.01	0.908
	NDVI	+	1	18.31	0.8	0.38
	Altitude	−	1	19.53	0.007	0.93
	Predator richness	−	1	18.10	0.01	0.906

The effect indicates the direction of the relationship (positive or negative). Significant effects are in bold. TL, total length; NDVI, normalized difference vegetation index.

### Variation of yellow pattern under common garden conditions

**Figure 3:**
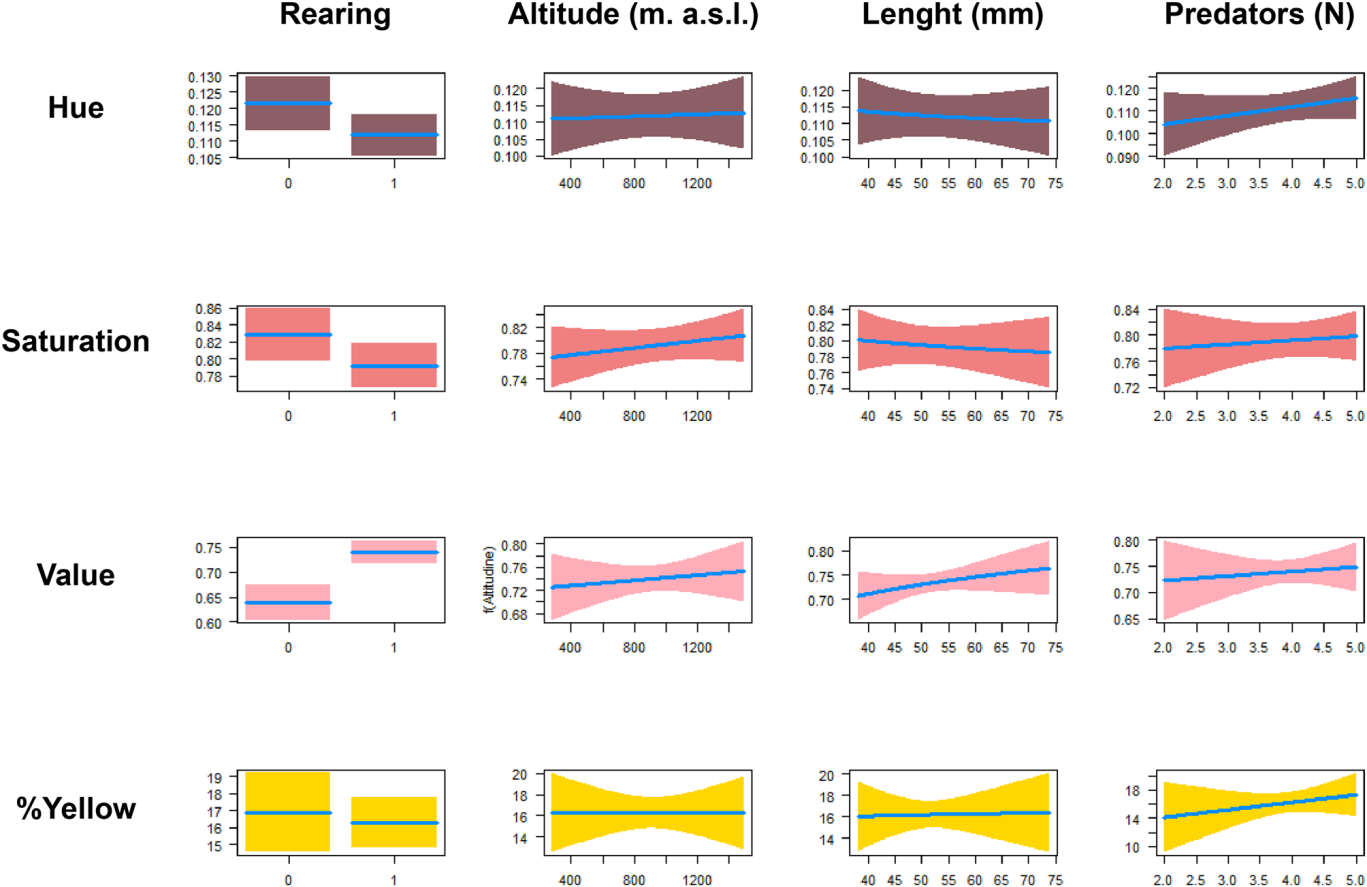
Conditional regression plots showing the results of univariate models (LMMs) assessing effect of fixed factors on components of the yellow coloration. Coloration components are tonality (Hue), Saturation, brightness (Value) and the percentage of yellow coverage. The effect of the rearing condition (nutritional regime), the altitudinal range and the length of the salamander at the metamorphosis and the number of predator species are shown (for a complete description of univariate model results see Table 2). The blue line represents the estimated median, while grey areas are 95% confidence bands.

Under rich food conditions, larvae took on average 78.7 ± 2.7 days to metamorphose, while under poor food conditions they required 362.3 ± 5.4 days. In total, 72 larvae (45 reared under rich food condition and 27 under poor food condition) reached metamorphosis and were photographed 45 days after metamorphosis. The average length of 45-day old metamorphs was 55.12 ± 1.14 mm. Their average percentage of dorsal yellow coloration was 16.75% ± 0.50 and ranged between 28.75 and 6.67%; averages (± SE) of color attributes were 0.11 ± 0.01 for Hue, 0.79 ± 0.05 for Saturation and 0.70 ± 0.01 for Value. Hue ranged between 0.14 and 0.08, Saturation between 0.89 and 0.60 while value between 0.84 and 0.51. The amount of trophic resources provided during larval stage was the only factor affecting coloration of metamporphs (MANOVA: Pillai’s trace = 0.48, *P* < 0.0001; Table 3), while we did not detect significant differences among larvae from different populations (MANOVA: Pillai’s trace = 0.66, *P* = 0.074; Table 3). The subsequent MANOVA including site-level features as dependent variables confirmed the effect of trophic features, and the lack of effect of any site-level variable (MANOVA: Pillai’s trace = 0.93, *P* = 0.15). Subsequent univariate analyses showed that metamorphs reared under rich food conditions have significantly lower hue and significantly higher saturation and value than larvae reared under poor food condition (Table 4). The coloration of metamorphs was unrelated to any site-level variable, nor to their total length (Table 4, Fig. 3).

**Table 3:** Multivariate analysis of variance (MANOVA) assessing the factors determining the variation of salamander’s dorsal pattern (hue, saturation, value and percentage of yellow) in neometamorphosed salamanders reared under different nutritional regimes.

Variable	Pillai's trace	NumDF	DenDF	F	*P*
**Between sites**					
Nutritional regime	0.66	4	7	3.42	0.074
Altitude	0.39	4	7	1.12	0.417
TL	0.28	4	7	0.70	0.614
Predators	0.23	4	7	0.52	0.718
**Within site**					
Nutritional regime	0.48	4	50	11.74	** < 0.001**
TL	0.03	4	50	0.49	0.735
Predators	0.06		50	0.86	0.493

The MANOVA included population identity as random factor, therefore distinguished between factors determining differences between and within population. Significant values are in bold. TL, total length.

**Table 4:** Results of the univariate analyses for the rearing experiment, as an independent factor we kept one of the components of yellow (percentage of yellow, hue, saturation and value) and as a dependent variable total length (TL), NDVI, altitude of origin site, the number of predators of the site the breeding condition.

Pattern	Effect	Estimate	NumDF	DenDF	F	*P*
Percentage of yellow	Rich food	−	1	64.53	0.10	0.749
	Altitude	+	1	21.09	0.0007	0.979
	TL	+	1	65.94	0.04	0.828
	Predators	+		29.04	0.79	0.379
Hue	Rich food	−	1	63.59	5.66	**0.020**
	Altitude	+	1	24.81	0.017	0.897
	TL	−	1	65.34	0.10	0.746
	Predators	+	1	34.81	1.13	0.293
Saturation	Rich food	−	1	65.24	4.35	**0.040**
	Altitude	+	1	35.06	0.67	0.415
	TL	−	1	65.93	0.16	0.685
	Predators	+	1	43.52	0.15	0.697
Value	Rich food	+	1	66	16.51	** < 0.001**
	Altitude	+	1	66	0.26	0.606
	TL	+	1	66	1.19	0.279
	Predators	+	1	66	0.18	0.666

Univariate linear mixed models (LMMs) assessing the factors determining the variation of components of salamander’s dorsal pattern in neometamorphosed salamanders reared under different food regimes. The effect indicates the direction of the relationship (positive or negative). Significant effects are in bold. TL, total length; NDVI, normalized difference vegetation index.

## Discussion

The aposematic coloration of fire salamanders showed significant variation (especially in the hue component) even between nearby populations. If color variation was mainly driven by selective forces, we expected some consistency in the variation of common-garden reared salamander and wild-caught adults, while if variation was mostly due to plasticity, we predicted that color variation under common-garden conditions was independent on population of origin. Our results provide low support for local adaptations, while the strong relationship between chromatic variation and environmental conditions appears to be mostly driven by phenotypic plasticity. For instance, the trophic availability experienced during early life stages seems to produce extremely strong effects on the aposematic pattern of newly metamorphosed salamanders, with juveniles experiencing rich food conditions showing a redder and more brilliant coloration than the ones experiencing poor nutritional regimes.

The significant difference in color patterns between populations conforms with the idea that evolutionary, ecological and physiological processes can concur in determining intraspecific variation of aposematism, still only part of our hypotheses on the potential drivers of this variation (ecosystem productivity and food availability; predator diversity; intrinsic features of individuals) were confirmed. Contrary to our expectation, we did not detect any relationship between the aposematic dorsal coloration and the richness of predators or altitude. Nevertheless, available data only showed a minimum variation of predator richness across different sites. In the Alps, predatory vertebrates tend to attain lower abundances at high altitudes^52^, and in absence of direct quantitative data on the abundance of predators, altitude can be considered as an additional proxy of overall variation of biotic communities (i.e., lower predator abundance and prey availability). Although a recent study showed that the relative size of the yellow pattern affects the probability of being predated, with less predation on individuals with more yellow coverage^30^ there is no evidence that as yellow increases also the toxicity of the animal raises^53^. Unfortunately, extensive, and accurate data on predation pressures on fire salamander population are missing, and our measures of predator presence were rough proxies, which were far to be comprehensive and likely dampened the robustness of predator effect assessment. For instance, more direct data on the actual abundance of the predators in the study populations (e.g. obtained directly using clay models and camera traps) can provide more robust conclusions. Finally, coloration expression can also depend on complex trade-offs in response to predator abundance and diversity (e.g., different predator species may react differently to salamander color patterns), further complicating the identification of drivers of aposematic coloration under different predation pressure regimes^30,53^. This fact calls for caution when assessing aposematic variation in response to predation and highlights the need of more focused studies on this topic.

Conversely, we found a clear relationship between color pattern and two parameters representing ecosystem productivity and prey availability, i.e., NDVI and abundance of earthworm droppings. This result matches the prediction that forests with higher productivity and with higher prey availability can sustain a larger trophic web with higher prey richness^54,55^. In turn, higher resource richness can foster the availability of costly pigments, such as carotenoids, which determine the yellow pattern of aposematic amphibians^56^. However, as the ecological meaning of the color differences recorded are unknown, further studies should disentangle the relative role of pigment production and gathering.

Beside the effect of environmental variation, we also found a significant relationship between color pattern and body size, while we did not detect differences between males and females. Recent studies showed that males can have more saturated yellow patterns than females^57^. This phenomenon has been interpreted as an intraspecific signal favoring male attraction during mates^57^. In our study case, even if sex played a significant effect in determining the overall color differences across populations, it was never significant in univariate analyses. This discrepancy could be caused by differences in the sex ratio of captured individuals between populations, and could also be related to the fact that we performed sampling in autumn, when males are more active than females^37^, thus in some populations we obtained a skewed sex ratio. The effect of body size, with smaller individuals showing higher proportion of yellow coloration, suggests a stronger aposematic pattern in young individuals. Fire salamanders show continuous body growth; thus, body size can be considered a proxy of the age of the animals. The fact that smaller and thus likely younger individuals tend to be yellower suggests that predation pressure could be stronger in the early phases after metamorphosis. This observation is confirmed by studies showing that the yellow pattern of adult fire salamander can change through time, with a reduction of the amount of yellow and the split of yellow spots^33^.

To disentangle the role of local adaptation versus plasticity in determining the color differences between population, we reared larvae under common garden conditions under different dietary regimes. This experiment showed a strong relationship between color pattern and diet, while under common rearing differences between populations vanished. Larvae that experienced rich food conditions showed lower hue (i.e. a warmer tone of yellow), higher saturation and higher value after metamorphosis. All these parameters indicate a brighter coloration compared to conspecifics which have eaten less during larval phase and underline that environmental conditions play a major role in affecting post-metamorphosis coloration of aposematic salamanders.

Aposematic coloration mainly has defensive roles; in fact, it reduces predatory risk and offers a major adaptive advantage to the grip, thus enhancing individual survival^58^. The study of species coloration and factors affecting that influence its expression and evolution is therefore extremely important to understand the biology and ecology of organisms that have striking colors^59,60^. Theory predicts that more fixed are aposematic coloration patterns, the easier it is for the predators to learn/recognize them^61,62^. In fire salamanders the overall aposematic pattern differs among the distinct phylogenetic lineages that occur across Europe^63,64^. Our study considered only one single salamander lineage in order to evaluate the relative importance of fine-scale local adaptations versus plasticity, and we found limited support for the possibility of local adaptations for the aposematic pattern, suggesting that variation is mostly explained by variability of resources between populations inhabiting different environments. However, across the species’ range fire salamanders show many eco-types easily distinguishable from their coloration pattern, suggesting that comparing populations over broader spatial or phylogenetic scales could reveal mechanisms related to genetic features of populations.

Despite the considerable interest on the mechanisms determining animal coloration, it is often very difficult to discern the factors influencing such a complex variable, because both extrinsic factors (environmental), intrinsic factors (physiology, age, sex) and local adaptations may interact in the development of aposematic colorations^65,66^. In fire salamanders, the strongest investment on the aposematic pattern seem to occurs in young individuals and is heavily affected by nutritional conditions experienced during larval development. These results are supported by the fact that the yellow pattern starts to develop in the larval stages short before metamorphosis^67^ and that younger individuals are likely the yellower individuals in the populations. Our study stresses the importance of plasticity and resource availability on the expression of aposematic coloration, and highlights the importance of jointly considering multiple processes, from local adaptations to resource availability, to identify the drivers of vertebrate color patterns.

## Supplementary Information

Supplementary Information 1.

Supplementary Information 2.

Supplementary Information 3.

## Data Availability

All data generated or analysed during this study are included in this published article and its supplementary information files.
