# Supplementary material for: Factors determining the dorsal coloration pattern of aposematic salamanders
Source: Sci Rep. 2022 Oct 12;12:17090. doi: 10.1038/s41598-022-19466-0 (PMC9556531; doi:10.1038/s41598-022-19466-0)
Supplement: Supplementary file 1 — Supplementary Information 1. [file 41598_2022_19466_MOESM1_ESM.docx]

| Site | Code | District | Altitude (m a.s. l.) | Breeding Site | Latitude and Longitude |
| --- | --- | --- | --- | --- | --- |
| Aizurro | AIZ | LC | 546 | Stream | 45°45'31.4"N 9°24'32.6"E |
| Bulciago | BUL | LC | 324 | Stream | 45°45'35.5"N 9°17'52.7"E |
| Civate | CIV | LC | 377 | Spring | 45°49'57.2"N 9°19'31.5"E |
| Colle Brianza | CLL | LC | 460. | Spring | 45°46'13.9"N 9°21'42.0"E |
| San Michele | SM | LC | 340 | Spring | 45°50'13.5"N 9°23'19.6"E |
| Val del Faè | FAE | LC | 610 | Spring | 45°50'11.2"N 9°22'42.1"E |
| Zizzanorre | ZIZ | LC | 330 | Spring | 45°44'06.8"N 9°17'48.0"E |
| Magreglio | MAG | CO | 944 | Spring | 45°55'16.5"N 9°14'56.0"E |
| Becogne | BEC | CO | 296 | Stream | 45°43'51.4''N 9°13'45.1''E |
| Pampello | PAM | CO | 285 | Spring | 45°44'38.8''N 9°14'27.5''E |
| Cosia | R3 | CO | 953 | Stream | 45°49'47.6"N 9°11'14.0"E |
| Affluente Cosia | R4 | CO | 930 | Stream | 45°50'02.1"N 9°11'10.1"E |
| Briosco | G8 | MB | 279 | Spring | 45°42'01.3"N 9°14'35.0"E |
| Valzurio Alta | ZURA | BG | 1491 | Spring | 45°56'22.5"N 9°59'08.3"E |
| Valzurio Bassa | ZURB | BG | 1200 | Spring | 45°55'55.2"N 9°59'04.4"E |

*Supplementary Table S1* – Sampling sites. The table shows the sites where both larvae and adults were sampled
